# Influence of Self-Determination and Social Support on Post-Traumatic Growth among Living Kidney Donors: A Cross-Sectional Study

**DOI:** 10.3390/medicina58091155

**Published:** 2022-08-25

**Authors:** Younghui Hwang, Kyoungok Min, Jihyun Oh

**Affiliations:** 1 Department of Nursing, College of Medicine, University of Ulsan, Ulsan 44610, Korea; 2 Department of Transplant Center, Seoul National University Hospital, Seoul 03080, Korea; 3 Department of Nursing, College of Nursing and Health, Kongju National University, Kongju 32588, Korea

**Keywords:** kidney donor, post-traumatic growth, self-determination, social support

## Abstract

*Background and Objectives*: Although many studies have reported that kidney donation is not physically harmful to living kidney donors, there are few studies on the psychological changes that they experience, especially post-traumatic growth. This study aimed to investigate the influence of self-determination and social support on post-traumatic growth among living kidney donors. *Materials and Methods*: This study used a descriptive, cross-sectional design. Data were collected from 114 living kidney donors who visited the outpatient solid organ transplant center at Seoul National University Hospital. The data were analyzed using the t-test, one-way analysis of variance, and stepwise multiple regression. *Results*: The results showed that the mean for post-traumatic growth of living kidney donors was 4.24 (0.81), a level higher than the midpoint. The factors affecting total post-traumatic growth were the relatedness of self-determination, the social support of their significant other, and donor type. In particular, the relatedness of self-determination was a significant factor affecting changed perceptions of self, relating to others, and spiritual change, subscales of post-traumatic growth. Additionally, the social support of donors’ significant others was a significant factor affecting relating to others and new possibilities, subscales of post-traumatic growth. *Conclusions*: Healthcare providers should endeavor to help living kidney donors experience post-traumatic growth, which can be facilitated by improving their self-determination and social support.

## 1. Introduction

Kidney donation is an important life-saving method for patients with end-stage renal disease, with living kidney transplantation accounting for nearly 40% of overall kidney transplantation activity [1,2]. Compared to the number of patients with end-stage renal disease who require kidney transplantation, the number of deceased donors is insufficient; thus, living kidney transplantation is actively being performed as an alternative in South Korea, and accounts for more than half of all kidney transplantations [3].

Organ trafficking is strictly prohibited by the Organ Transplantation Act, and living kidney donations are guaranteed by the same act so that they can be voluntarily carried out. Voluntariness and self-determination are important in the legal and ethical frameworks of living kidney donation. For kidney donations not to be determined or manipulated by coercion, the process must be performed by the autonomous and voluntary behavior of the living kidney donor [4]. According to self-determination theory, self-determination behavior can be achieved when basic psychological needs, such as autonomy, competence, and relatedness are satisfied; thus, it is important to possess autonomy in social relationships [5]. In addition, self-determination behavior is essential for facilitating optimal functioning for growth and integration, as well as for personal well-being [5]. Therefore, it can be predicted that the self-determination behavior of living kidney donors is an important factor in their mental growth and well-being.

Donating a kidney is a life-changing experience [6], and living kidney donors may experience physical, psychological, and social difficulties, such as pain, shock, anxiety, depression, uncertainty about its impact on their health, and financial problems [7]. However, a recent Kidney Donor Outcome Cohort study found that there were no incremental changes in psychosocial outcomes over time after donation, such as mood disturbance, body image concerns, fear of kidney failure, and life satisfaction; rather, living kidney donors developed social functioning from pre- to post-donation [8]. In other words, even if a living kidney donor experiences post-traumatic stress during the donation process [6,7], it can be predicted that living kidney donors will recognize the donation process as a positive event rather than a negative event. If wisdom is created through positive post-traumatic experiences and self-reflection, post-traumatic growth can be achieved [9]. Therefore, kidney donors may experience post-traumatic growth through kidney donation [10]. 

As motivation induces personal growth and self-conceptualization, according to self-determination theory, autonomous motivation can lead to positive cognitive, behavioral, and affective outcomes [11]. Living kidney donors’ self-determination can affect not only donation decisions but also the post-traumatic growth that recognizes and responds to the post-donation process and outcomes. 

Social support refers to all forms of help exchanged in stable interpersonal relationships. Patients with high social support experience higher post-traumatic growth because they can express their thoughts and feelings more often in stable interpersonal relationships [12]. In other words, social support can give rise to post-traumatic growth by helping patients to increase interaction through stable interpersonal relationships and finding new perspectives and positive meanings for life [13]. Therefore, high social support among living kidney donors is expected to have a positive effect on post-traumatic growth.

Yucetin et al. [10] reported that there were differences in post-traumatic growth depending on the education level, marital status, and age of living kidney donors. Thus, it can be predicted that the general characteristics of living kidney donors will also affect post-traumatic growth.

However, few studies have investigated the effects of living kidney donors’ general characteristics, self-determination, and social support on post-traumatic growth. Investigating the effects of these factors on post-traumatic growth may help develop interventions in which kidney donors can grow mentally through the kidney donation process. Therefore, this study investigated the effects of living kidney donors’ general characteristics, self-determination, and social support on post-traumatic growth.

## 2. Materials and Methods

### 2.1. Study Design and Participants

This study used a cross-sectional and descriptive design to investigate the factors affecting post-traumatic growth of living kidney donors in South Korea. Participants were living kidney donors who visited the outpatient solid organ transplant center at Seoul National University Hospital for regular post-donation medical physical examinations between 1 November 2019 and 15 August 2020. The participants were adults aged ≥18 years who understood the purpose of the study, agreed to participate in the study, and were able to understand and respond to the questionnaire. The required sample size was calculated using G Power 3.1.9.7 (G*Power, Düsseldorf, Germany) [14]. The minimal sample size (*n* = 98) was calculated under the assumptions of a significance level of 0.05, an effect size of 0.15, and a power of 0.80. A total of 120 living kidney donors agreed to participate in the study; however, 114 living kidney donors completed the questionnaire. All questionnaires were distributed by personal contact, exercising appropriate precautions during the COVID-19 pandemic. The overall response was therefore 95%. Thus, data from 114 participants (a suitable sample size) were included in the final analysis.

### 2.2. General Characteristics

Participants’ general characteristics were examined by age, sex, education, marital status, religion, donor type, donation recommendation, health recovery after donation, and autonomy of donation decision.

### 2.3. Post-Traumatic Growth

The Post-Traumatic Growth Inventory (PTGI) developed by Tedeschi and Calhoun [15], translated into Korean and validated by Song and Lee [16], was used to assess positive outcomes after experiencing a traumatic event. The K-PTGI consists of 16 items across 4 subscales: change in relationships with others, identifying new possibilities, change in self-perception, and spiritual change. The items were scored on a 6-point Likert scale ranging from 1 (experienced no change) to 6 (experienced a great degree of change). The total scores ranged from 16 to 96, with higher scores indicating greater levels of post-traumatic growth. Cronbach’s α was 0.90 in the original PTGI development and was 0.88 for the overall scale in this study. Cronbach’s α values were calculated for change in relationships with others (Cronbach’s α = 0.85), identifying new possibilities (Cronbach’s α = 0.87), change in self-perception (Cronbach’s α = 0.90), and spiritual change (Cronbach’s α = 0.70).

### 2.4. Multidimensional Scale of Perceived Social Support

The Multidimensional Scale of Perceived Social Support (MPSS), developed by Zimet and Dahlem [17], was used to estimate the perceived adequacy of social support. The MPSS measures the adequacy of social support from three categories of people: significant other support, family support, and friend support. The 12-item MPSS is rated on a 7-point Likert scale ranging from ‘very strongly disagree’ (score 1) to ‘very strongly agree’ (score 7). The total scores ranged from 12 to 84, with higher scores representing higher levels of perceived social support. In this study, Cronbach’s α for the overall scale was 0.95. Cronbach’s α was 0.94 for the significant other support subscale, 0.89 for the family support subscale, and 0.85 for the friend support subscale. 

### 2.5. Self-Determination

Self-determination was measured using the Korean version of the Basic Psychological Needs Index developed by Deci and Ryan [18], which was translated, modified, and verified for reliability and validity by Lee and Kim [19]. This scale consists of 18 questions that measure autonomy (regulating the autonomy of behavior), competence (fully using one’s ability), and relatedness (interacting with others in a social context). This tool was measured on a 5-point Likert scale (from 1 to 5); the higher the score, the higher the degree of self-determination. Cronbach’s alphas were 0.87 for Lee and Kim’s study [19] and 0.90 for the total scale in this study, indicating high reliability. The autonomy, competence, and relatedness subscales for this study had Cronbach’s α values of 0.78, 0.84 and 0.81, respectively. 

### 2.6. Data Collection

The study was conducted between 1 November 2019 and 15 August 2020, at the outpatient solid organ transplant center at Seoul National University Hospital. Data were collected from living kidney donors by using structured questionnaires and medical charts. The researchers explained the purpose of the study and distributed the questionnaires to the participants. The subjects agreed to participate prior to returning the completed questionnaires to the researchers. All participants were given a small gift of gratitude for their voluntary participation.

### 2.7. Ethical Considerations

This study was approved by the Institutional Review Board of Seoul National University (IRB No: H-1909-151-1067, approved on 23 October 2019). The participants were enrolled only after they provided written informed consent. In addition, participants were informed that they could choose not to participate in the study at any time without any personal disadvantage or penalty. All self-questionnaires were completed anonymously and data on each participant’s information were coded. The collected data were maintained in the researchers’ private office, which was locked to ensure confidentiality.

### 2.8. Statistical Analysis

Data were analyzed using SPSS statistical software (version 26.0; IBM Corp, Armonk, NY, USA). Participants’ general characteristics, post-traumatic growth, the multidimensional scale of perceived social support, and self-determination were analyzed using descriptive statistics (frequency and percentage). Differences in post-traumatic growth according to the general characteristics were analyzed using the independent t-test and one-way ANOVA, and the Scheffé post-test was used. Correlations among post-traumatic growth, the multidimensional scale of perceived social support, and self-determination were examined using Pearson’s correlation coefficients. Stepwise multiple regression analysis was performed to analyze factors affecting post-traumatic growth. 

## 3. Results

### 3.1. General Characteristics of the Participants and Post-Traumatic Growth according to General Characteristics

Out of the 120 participants, 114 fully participated in the study (response rate: 95%) (Table 1). The mean age of the participants was 54.4 years (SD = 10.1), ranging from 24 to 78 years, and 37.7% of the subjects were 51–60 years old. More than half of the participants were men (52.6%), and 86.8% were married. Concerning educational level, 50.9% had a high school education or higher. A majority of participants (62.3%) were affiliated with religious groups. The relationships between kidney donors and recipients were mostly family members (59.6%), and 84.2% of the participants answered ‘yes’ when asked if they would recommend donation to others. Most participants (81.6%) recovered from their health condition after donation, and the donation decision was made voluntarily (100%).

Regarding the total post-traumatic growth scores according to the participants’ general characteristics, there was a significant difference in gender (t = −2.398, *p* = 0.018), religion (t = 2.026, *p* = 0.045), and donor type (F = 4.549, *p* = 0.013). 

In the subdomains of post-traumatic growth, regarding differences in change in self-perception according to participants’ general characteristics, there were significant differences in gender (t = −3.067, *p* = 0.003) and type of donor (F = 7.158, *p* = 0.001). The results of the post hoc tests with ANOVA showed that spousal donors showed significantly higher changes in self-perception scores than family donors and non-blood relationship donors. Regarding changes in relationships with others according to the participants’ general characteristics, there were significant differences depending on the type of donor (F = 3.290, *p* = 0.041) and donation recommendation (t = 2.372, *p* = 0.010). Regarding spiritual changes according to the participants’ general characteristics, there were significant differences in age group (F = 4.418, *p* = 0.014), gender (t = −3.982, *p* < 0.001), marital status (t = −2.120, *p* = 0.036), religion (t = 6.191, *p* < 0.001), and type of donor (F = 4.505, *p* = 0.013). Post hoc testing showed that the spousal donors showed significantly higher spiritual changes than the family donors. 

### 3.2. Levels of Post-Traumatic Growth, Multidimensional Scale of Perceived Social Support, and Self-Determination

The levels of post-traumatic growth, the multidimensional scale of perceived social support, and self-determination are presented in Table 2. The total mean score for post-traumatic growth was 4.24 (SD = 0.81) out of the maximum possible score of 6. Of the post-traumatic growth subscales, the mean scores for change in relationships with others, change in self-perception, identifying new possibilities, and spiritual change were 4.23 (SD = 0.83), 4.15 (SD = 1.07), 4.48 (SD = 0.82), and 3.75 (SD = 1.38), respectively. Of the social support subscales, the mean scores for family support, friend support, and significant other support were 5.90 (SD = 0.92), 5.30 (SD = 1.09), and 5.10 (SD = 1.48), respectively. Of the self-determination subscales, autonomy showed the highest score (M = 3.99, SD = 0.55), followed by relatedness (M = 3.87, SD = 0.52) and competence (M = 3.67, SD = 0.55). 

### 3.3. Correlations between Post-Traumatic Growth, Multidimensional Scale of Perceived Social Support, and Self-Determination 

The correlations among the variables are presented in Table 3. 

### 3.4. Factors Affecting Post-Traumatic Growth

The factors affecting post-traumatic growth are listed in Table 4. The most powerful factor affecting the total post-traumatic growth was the relatedness of self-determination (β = 0.468, *p* = 0.001). The next most powerful factor affecting post-traumatic growth was the type of donor (β = 0.288, *p* = 0.032), followed by the social support of donors’ significant others (β = 0.134, *p* = 0.007). The explanatory power of these variables was 19.5% (F = 10.041, *p* < 0.001). The variance inflation factor values were all less than 10, with no multicollinearity, and the Durbin–Watson value was sufficient to satisfy the independence of residuals (Table 4).

Factors affecting higher changes in self-perception scores of post-traumatic growth were the relatedness of self-determination (β = 0.363, *p* = 0.017), spousal donor of donor type (β = 0.313, *p* = 0.020), female gender (β = 0.307, *p* = 0.032), and family support (β = 0.224, *p* = 0.010). Factors affecting changes in relationships with others’ scores of post-traumatic growth were more significant social support of donors’ significant others (β = 0.149, *p* = 0.003) and the relatedness of self-determination (β = 0.480, *p* = 0.001). Additionally, factors affecting a higher identification of new possible scores of post-traumatic growth were more highly significant for social support of donors’ significant others (β = 0.182, *p* = 0.008). Factors affecting higher spiritual change scores of post-traumatic growth were higher relatedness of self-determination (β = 0.569, *p* = 0.006), female gender (β = 0.553, *p* = 0.012), religion (β = 1.171, *p* < 0.001), and spousal donor of donor type (β = 0.567, *p* = 0.006). This explained variance ranging from 5.4% (identifying new possibilities) to 36.9% (spiritual changes).

## 4. Discussion

This study evaluated the self-determination, social support, and post-traumatic growth of living kidney donors in Korea and the effect of self-determination and social support on post-traumatic growth.

In this study, the mean for post-traumatic growth of living kidney donors was 4.24 (0.81) out of 6, a level higher than the midpoint. This score is higher than the average score measured for post-traumatic growth in patients with breast cancer [12] and kidney transplant recipients [20] using the same tool. This result shows that living kidney donors experienced moderate-to-high levels of post-traumatic growth and more post-traumatic growth than previously reported for cancer patients and kidney transplant recipients, which is consistent with the results of a previous study [10]. Among the subscales of post-traumatic growth, the mean score of change in self-perception was the highest, and the mean score of spiritual change was the lowest, similar to findings in living kidney donors [21]. However, in order to understand how living kidney donors experience post-traumatic growth through kidney donation, a longitudinal study should be conducted to understand changes in the post-traumatic growth of kidney donors before and after kidney donation.

A novel finding of this study was that the main factor affecting post-traumatic growth was the relatedness of self-determination. In self-determination theory, relatedness is one of the basic psychological needs that leads to self-determination by inducing intrinsic and extrinsic motivations, along with autonomy and competence as feelings of belongingness and connectedness with others [5]. This relatedness allows non-selfish autonomy and competence in social relationships [5]. In other words, living donors with a strong relatedness were able to make a willing decision to donate their kidneys for the health of the recipients who were meaningful to them. As donation is an act of self-determination, it can be assumed that they positively recognized and accepted various psychological and social problems that occur during the donation process or after donation, and therefore further experienced post-traumatic growth [10]. In addition, the relatedness of self-determination had the most influence on change in self-perception, change in relationships with others, and spiritual change among the subcategories of post-traumatic growth. This result shows that donors with strong relatedness can develop post-traumatic growth in their perception of themselves, relationships with others, and spirituality. The self-determination of living donors is a very important factor that can affect the psychological and social problems living donors may experience during the donation process or after donation during post-traumatic growth. Therefore, future research should be conducted to develop a strategy that can increase the self-determination of the post-traumatic growth of living kidney donors.

Donor type is an additional factor that was found to influence post-traumatic growth, and living kidney donors who donated their kidneys to their spouses (spouse donors) experienced more post-traumatic growth than living kidney donors who donated their kidneys to their families. Spouse donors can have a higher partnership satisfaction with the kidney recipient compared to non-spouse donors, and this high partnership satisfaction can have a positive effect on the body and mind of living kidney donors [22]. However, the donor–recipient relationship, which may be improved through their ability to participate in life together, is an unstable relationship that can be greatly affected by behavioral and psychological changes in recipients [23]; thus, further research will be needed on spouses’ experiences of post-traumatic growth after kidney donation. 

It is also important to note that social support, especially from other living donors and healthcare providers, affects living donors’ post-traumatic growth. Social support may affect the post-traumatic growth of living kidney donors because social networks may mitigate stress and provide donors with opportunities to adopt a new view associated with increased growth [13]. Information and psychological support provided by healthcare providers and other living kidney donors can help donors actively cope with the various difficulties related to donation and can provide emotional stability [24]. Therefore, healthcare providers should provide opportunities for kidney donors to share the suffering associated with kidney donation and to rely on other kidney donors with similar experiences, and should also provide various information and psychological support, so that living kidney donors can experience post-traumatic growth.

In this study, living kidney donors experienced post-traumatic growth, and the factors affecting post-traumatic growth were the relatedness of self-determination, donation type (spouse donor), and social support. Therefore, it will be helpful to use strategies that increase the relatedness of self-determination and social support when developing an intervention program in which living kidney donors can experience post-traumatic growth.

This study had several limitations. First, the generalization of the research results requires caution, as the participants were living kidney donors who visited a university hospital. Next, because this study investigated the post-traumatic growth of living kidney donors using a cross-sectional method, continuous changes in post-traumatic growth could not be identified. In addition to self-determination and social support, there may be additional variables that affect post-traumatic growth, but these were not considered in this study.

## 5. Conclusions

This study suggests that living kidney donors experience post-traumatic growth, and that self-determination and social support affect post-traumatic growth. These results show that various physical and psychological problems that can occur in living kidney donors can be solved using post-traumatic growth. Therefore, health care providers should provide various types of information and psychological support to kidney donors, and help the relatedness of self-determination of living kidney donors to become sufficient enough so that living kidney donors can experience post-traumatic growth. 

## Figures and Tables

**Table 1:** Participants’ general characteristics and post-traumatic growth according to general characteristics (*n* = 114).

Characteristics	Mean	Range	*n* (%)	Post-Traumatic Growth Inventory				
				Total	Change in Self-Perception	Change in Relationships with Others	Identifying New Possibilities	Spiritual Change
				Mean (SD)				
Age (year)	54.4 (10.1)	24–78						
≤50 ^a^			36 (31.6)	4.11 (0.84)	4.35 (0.85)	4.15 (0.79)	4.00 (1.23)	3.45 (1.36)
51–60 ^b^			43 (37.7)	4.23 (0.76)	4.53 (0.75)	4.27 (0.83)	4.10 (1.04)	3.54 (1.40)
≥60 ^c^			35 (30.7)	4.40 (0.85)	4.55 (0.89)	4.26 (0.89)	4.38 (0.93)	4.31 (1.22)
*F* *(p)*				1.117 (0.331)	0.641 (0.529)	0.237 (0.789)	4.15 (1.07)	4.418 (0.014) a,b < c
Sex								
Male			60 (52.6)	4.07 (0.88)	4.26 (0.88)	4.13 (0.87)	4.17 (1.15)	3.29 (1.37)
Female			54 (47.4)	4.44 (0.69)	4.72 (0.68)	4.34 (0.78)	4.13 (1.00)	4.27 (1.20)
t(*p*)				−2.398 (0.018)	−3.067 (0.003)	−1.337 (0.184)	0.166 (0.869)	−3.982 (<0.001)
Education								
≤Elementary school			5 (4.4)	4.30 (0.92)	4.83 (0.63)	4.28 (1.00)	3.80 (1.67)	4.37 (1.49)
Middle school			51 (44.7)	4.19 (0.78)	4.40 (0.84)	4.19 (0.81)	4.22 (0.96)	3.58 (1.31)
≥High school			58 (50.9)	4.28 (0.84)	4.52 (0.83)	4.26 (0.85)	4.13 (1.12)	3.87 (1.43)
*F(p)*				0.167 (0.847)	0.751 (0.474)	0.119 (0.888)	0.370 (0.692)	1.009 (0.368)
Marital status								
Single/other			15 (13.2)	3.97 (1.09)	4.28 (1.03)	3.94 (1.14)	4.00 (1.30)	3.06 (1.92)
Married			99 (86.8)	4.29 (0.76)	4.51 (0.79)	4.27 (0.77)	4.18 (1.04)	3.86 (1.25)
t(*p*)				−1.400 (0.164)	−0.962 (0.338)	−1.440 (0.153)	−0.601 (0.549)	−2.120 (0.036)
Religion								
Yes			71 (62.3)	4.36 (0.76)	4.55 (0.79)	4.26 (0.82)	4.24 (1.04)	4.30 (1.19)
No			43 (37.7)	4.04 (0.87)	4.35 (0.88)	4.18 (0.86)	4.00 (1.12)	2.85 (1.19)
t(*p*)				2.026 (0.045)	1.222 (0.224)	0.529 (0.598)	1.126 (0.263)	6.191 (<0.001)
Type of donor								
Family donor ^d^			68 (59.6)	4.10 (0.91)	4.33 (0.90)	4.15 (0.90)	3.99 (1.13)	3.44 (1.50)
Spouse donor ^e^			44 (38.6)	4.50 (0.55)	4.76 (0.55)	4.41 (0.68)	4.40 (0.97)	4.22 (1.05)
Unrelated donor (non-blood) ^f^			2 (1.8)	3.43 (0.17)	3.08 (0.82)	3.10 (0.42)	4.16 (0.23)	4.25 (0.35)
*F(p)*				4.549 (0.013)	7.158 (0.001) f < d < e	3.290 (0.041)	2.039 (0.135)	4.505 (0.013) d < e
Donation recommendation								
Yes			96 (84.2)	4.30 (0.82)	4.53 (0.82)	4.31 (0.84)	4.19 (1.08)	3.81 (1.33)
No			18 (15.8)	3.93 (0.73)	4.19 (0.81)	3.80 (0.66)	3.94 (1.07)	3.47 (1.64)
t(*p*)				1.740 (0.085)	1.545 (0.125)	2.372 (0.010)	0.891 (0.381)	0.935 (0.352)
Recovery after donation								
Yes			93 (81.6)	4.22 (0.83)	4.46 (0.83)	4.21 (0.84)	4.14 (1.03)	3.70 (1.37)
No			21 (18.4)	4.34 (0.76)	4.55 (0.80)	4.31 (0.79)	4.20 (1.29)	4.00 (1.39)
t(*p*)				−0.597 (0.552)	−0.456 (0.649)	−0.476 (0.635)	−0.234 (0.815)	−0.887 (0.377)
Donation decision								
Voluntary			114 (100)					

SD, standard deviation; a = lee than or equal to 50; b = between 51 and 60; c = greater than or equal to 61; d = family donor; e = spouse donor; f = unrelated donor.

**Table 2:** Scores for post-traumatic growth, social support, and self-determination (*n =* 114).

Variables	Range	Mean (SD)
Post-traumatic growth inventory	1.31–5.88	4.24 (0.81)
Change in relationships with others	1.0–5.8	4.23 (0.83)
Identifying new possibilities	1–6	4.15 (1.07)
Change in self-perception	1.67–6	4.48 (0.82)
Spiritual change	1–6	3.75 (1.38)
Social support		
Family support	1.75–7.0	5.90 (0.92)
Friend support	1.75–7.0	5.30 (1.09)
Significant other support	1.0–7.0	5.10 (1.48)
Self-determination		
Autonomy	2.5–5	3.99 (0.55)
Competence	2–5	3.67 (0.55)
Relatedness	2–5	3.87 (0.52)

SD, standard deviation.

**Table 3:** Correlations among variables (*n =* 114).

Variables	r (*p*)									
	Post-Traumatic Growth Inventory				Social Support			Self-Determination		
	1	2	3	4	5	6	7	8	9	10
1. Change in relationships with others	-									
2. Identifying new possibilities	0.665 (<0.001)	-								
3. Change in self-perception	0.868 (<0.001)	0.677 (<0.001)	-							
4. Spiritual change	0.489 (0.563)	0.413 (<0.001)	0.566 (<0.001)	-						
5. Family support	0.308 (0.001)	0.128 (0.178)	0.360 (<0.001)	0.166 (0.079)	-					
6. Friend support	0.293 (0.002)	0.056 (0.026)	0.271 (0.004)	0.121 (0.202)	0.579 (<0.001)	-				
7. Significant other support	0.346 (<0.001)	0.250 (0.008)	0.343 (<0.001)	0.188 (0.047)	0.483 (<0.001)	0.555 (<0.001)	-			
8. Autonomy	0.098 (0.302)	0.042 (0.661)	0.121 (0.203)	0.043 (0.655)	0.370 (<0.001)	0.303 (0.001)	0.269 (0.004)	-		
9. Competence	0.338 (<0.001)	0.168 (0.076)	0.308 (0.001)	0.163 (0.085)	0.431 (<0.001)	0.493 (<0.001)	0.361 (<0.001)	0.411 (<0.001)	-	
10. Relatedness	0.372 (<0.001)	0.161 (0.088)	0.352 (<0.001)	0.241 (0.011)	0.466 (<0.001)	0.645 (<0.001)	0.272 (0.004)	0.432 (<0.001)	0.651 (<0.001)	-

**Table 4:** Factors affecting post-traumatic growth (*n* = 114).

Variable	Post-Traumatic Growth				
	Total	Change In Self-Perception	Change in Relationships with Others	Identifying New Possibilities	Spiritual Change
	β (SE)				
Constant	1.338 (0.581) *p* = 0.023	0.849 (0.633) *p* = 0.183	1.611 (0.544) *p* = 0.004	3.226 (0.356) *p* = 0.000	1.542 (0.983) *p* = 0.120
Sex		0.307 (0.186) *p* = 0.032			0.553 (0.218) *p* = 0.012
Religion					1.171 (0.224) *p* < 0.001
Type of donor	0.288 (0.132) *p* = 0.032	0.313 (0.132) *p* = 0.020			0.567 (0.201) *p* = 0.006
Social support, Family		0.224 (0.085) *p* = 0.010			
Social support, Significant other	0.134 (0.243) *p* = 0.007		0.149 (0.050) *p* = 0.003	0.182 (0.067) *p* = 0.008	
Self-determination, Relatedness	0.468 (0.300) *p* = 0.001	0.363 (0.150) *p* = 0.017	0.480 (0.141) *p* = 0.001		0.569 (0.203) *p* = 0.006
R^2^	0.217	0.251	0.203	0.063	0.392
Adj. R^2^	0.195	0.224	0.188	0.054	0.369
F(*p*)	10.041 (*p* < 0.001)	5.577 (*p* = 0.020)	17.790 (*p* < 0.001)	7.414 (*p* = 0.008)	7.879 (*p* = 0.006)

SE, standard error.

## Data Availability

The data that support the findings of this study are available from the corresponding author.
